# Gene–environment interaction study on the polygenic risk score for neuroticism, childhood adversity, and parental bonding

**DOI:** 10.1017/pen.2023.2

**Published:** 2023-08-04

**Authors:** Boris Klingenberg, Sinan Guloksuz, Lotta-Katrin Pries, Ozan Cinar, Claudia Menne-Lothmann, Jeroen Decoster, Ruud van Winkel, Dina Collip, Philippe Delespaul, Marc De Hert, Catherine Derom, Evert Thiery, Nele Jacobs, Marieke Wichers, Bochao D. Lin, Jurjen Luykx, Jim van Os, Bart P. F. Rutten

**Affiliations:** 1 Department of Psychiatry and Neuropsychology, Maastricht University Medical Centre, The Netherlands; 2 Department of Psychiatry, Yale School of medicine, USA; 3 University Psychiatric Centre, KU Leuven, Belgium; 4 Antwerp Health Law and Ethics Chair, AHLEC University Antwerpen, Antwerp, Belgium; 5 Centre of Human Genetics, University Hospitals Leuven, Belgium; 6 Department of Obstetrics and Gynaecology, Ghent University Hospitals, Belgium; 7 Department of Neurology, Ghent University Hospitals, Belgium; 8 Faculty of Psychology and Educational Sciences, Open University of the Netherlands, The Netherlands; 9 Department of Psychiatry, University Medical Center Groningen, The Netherlands; 10 The Interdisciplinary Center Psychopathology and Emotion Regulation (ICPE), The Netherlands; 11 Brain Centre Rudolf Magnus, University Medical Center Utrecht, The Netherlands; 12 King’s College London, King’s Health Partners, Department of Psychosis Studies, Institute of Psychiatry, UK

**Keywords:** Endophenotype, Environment, Genetics, Neuroticism, Personality

## Abstract

The present study examines whether neuroticism is predicted by genetic vulnerability, summarized as polygenic risk score for neuroticism (PRS_*N*_), in interaction with bullying, parental bonding, and childhood adversity. Data were derived from a general population adolescent and young adult twin cohort. The final sample consisted of 202 monozygotic and 436 dizygotic twins and 319 twin pairs. The Short Eysenck Personality questionnaire was used to measure neuroticism. PRS_*N*_ was trained on the results from the Genetics of Personality Consortium (GPC) and United Kingdom Biobank (UKB) cohorts, yielding two different PRS_*N*_. Multilevel mixed-effects models were used to analyze the main and interacting associations of PRS_*N*_, childhood adversity, bullying, and parental bonding style with neuroticism. We found no evidence of gene–environment correlation. PRS_*N*_ thresholds of .005 and .2 were chosen, based on GPC and UKB datasets, respectively. After correction for confounders, all the individual variables were associated with the expression of neuroticism: both PRS_*N*_ from GPC and UKB, childhood adversity, maternal bonding, paternal bonding, and bullying in primary school and secondary school. However, the results indicated no evidence for gene–environment interaction in this cohort. These results suggest that genetic vulnerability on the one hand and negative life events (childhood adversity and bullying) and positive life events (optimal parental bonding) on the other represent noninteracting pathways to neuroticism.

One of the dimensions of personality with relevance to society and mental health is neuroticism (Cuijpers et al., 2010; Lahey, 2009). Neuroticism can be defined as elevated stress reactivity and tendency to strong experiences of negative emotions such as sadness, anxiety, and anger, thus representing a broad negative affective dysregulation phenotype (Barlow, Ellard, Sauer-Zavala, Bullis & Carl, 2014). Numerous questionnaires measure this construct, and a strong association between these measures has been shown in the literature consistently (Aluja, Garcia & Garcia, 2004; De Fruyt, Van de Wiele & Van Heeringen, 2000; Draycott & Kline, 1995 ; Larstone, Jang, Livesley, Vernon & Wolf, 2002; Waller, DeYoung & Bouchard, 2016). Furthermore, the negative emotionality of the “Multidimensional Personality Questionnaire” and harm avoidance subscales of the “Temperament and Character Inventory” correlate highly with the general phenotype of neuroticism, albeit with slight differences.

Neuroticism represents a complex trait and has been linked to various genetic, neuroanatomical, and functional brain regions and is influenced further by environmental effects. Recent methods assessing the underlying genetic mechanisms of neuroticism used large genome-wide meta-analysis with populations over 100,000 (Okbay et al., 2016; Smith et al., 2016). Okbay et al. identified 11 variants to be associated with neuroticism, of which two tagged inversion polymorphisms in chromosomes 8 and 17 (Okbay et al., 2016). A recent study combining epigenetic and genetic data integrated DNA methylation data (using life course consistent methylation quantitative trait loci meQTLs) and GWAS data and furthermore performed pathway analyses to identify biological pathways showing enrichment (Zhao & Liu, 2020). This study observed enrichment for genes with roles in 21 brain regions as well as in the reproductive and immune systems (Zhao & Liu, 2020). Neuroticism is a highly polygenic phenotype involving complex multifaceted neural (and likely also general physiological) circuitries and has differential utility in both population and clinical samples (Gottschalk & Domschke, 2017; Grasby et al., 2020).

Environmental factors that seem to shape neuroticism are early developmental trauma and stressful life events (SLEs), as well as social roles and normative life transitions (Gottschalk & Domschke, 2017; Specht et al., 2014). Furthermore, neuroticism is influenced by an abundance of environmental factors that have implications for disease (Bucher, Suzuki & Samuel, 2019; Lehto, Karlsson, Lundholm & Pedersen, 2019).

Although genetic and environmental effects are individually relevant, research on the interplay of genotype and environment in regard to personality formation and change is of primary interest. For instance, it has been shown that neuroticism is mediated by negative life events as well as that there is mediation by genetic effect on negative life events (Kandler, Bleidorn, Riemann, Angleitner & Spinath, 2012). These findings underline the importance of further fine grain analysis by assessing specific environmental risk and protective factors such as childhood adversity, bullying, or parental bonding. A genome-wide interaction study detected gene–environment interactions for one SNP (rs115385310) with the broadly defined environmental risk factor of: “Felt hated by a family member as a child” (Werme, van der Sluis, Posthuma & de Leeuw, 2021). A recent paper has posited an integrative model underlining the interplay between person-level variation and environmental variation (Wagner, Orth, Bleidorn, Hopwood & Kandler, 2020).

Using the strengths of GWAS and considering the polygenic character of neuroticism, polygenic risk score as a single genetic risk measure for neuroticism (PRS_*N*_) has given new insights, especially in regard to GxE studies. For instance, a recent twin study used a gene–environment model to show an interaction between PRS_*N*_ and rearing status (reared together versus apart) on depressive symptoms (Lehto et al., 2019). A study assessing prenatal maternal risk factors in relation to several PRS and child behavioral problems found that PRS_*N*_ only predicted child internalizing behavioral problems, maternal alcohol use, and maternal anxiety during pregnancy (Ensink et al., 2020). An added layer of challenge is that early SLEs, for instance bullying, are partially heritable and separately associated with underlying polygenic risk scores (Schoeler et al., 2019). Therefore, these gene–environment correlations also need to be taken into account.

In light of this background, we tested the contribution of gene–environment interaction to neuroticism in a unique general population twin cohort of young adults and adolescents with deep phenotyping. To elucidate the role of several key environmental factors on the development of neuroticism, we tested whether the molecular genetic risk score for neuroticism (PRS_*N*_) interacts with environmental factors (i.e., parental bonding, bullying and childhood adversity) to influence neuroticism.

## 1. Methods

### 1.1. Sample

Data were derived from the first wave of the TwinssCan, a general population twin cohort that started including adolescent and young adult (age range = 15–35 years) twins (*n* = 796), their siblings (*n* = 43), and parents (*n* = 363) from April 2010 to April 2014 (Pries et al., 2017; Pries, Klingenberg et al. 2020). The TwinssCan cohort comprises individuals fulfilling the inclusion criteria from the East Flanders Prospective Twin Survey (Derom et al., 2013; Derom et al., 2019), a prospective population-based, multi-birth registry positioned in Flanders, Belgium. Participants were excluded if they had a pervasive developmental disorder as indicated by caregivers. Sequential analysis based on sex, fetal membranes, umbilical cord blood groups, placental alkaline phosphatase, and DNA fingerprints was used to determine zygosity (Derom et al., 2013). All participants gave written informed consent, and parent(s) signed an informed consent for participants below the age of 18 years. The local ethics committee approved the study (Commissie Medische Ethiek van de Universitaire ziekenhuizen KU Leuven, Nr. B32220107766). The authors assert that all procedures contributing to this work comply with the ethical standards of the relevant national and institutional committees on human experimentation and with the Helsinki Declaration of 1975, as revised in 2008.

### 1.2. Environmental measures

#### 1.2.1. Childhood adversity

Childhood adversity was assessed with the Dutch translation of the short version of the Childhood Trauma Questionnaire (CTQ) (Bernstein, Ahluvalia, Pogge & Handelsman, 1997). The CTQ comprises of five subscales: sexual abuse, emotional abuse, physical abuse, physical neglect, and emotional neglect; totaling 28 items of which 3 are Minimization/Denial validity items and 5 items per subscale. Participants were asked to rate on a scale from 1 “never” to 5 “always.” The CTQ has been validated for the Dutch population showing a Cronbach’s alpha of .91 for the physical abuse scale, .89 for emotional abuse, .95 for sexual abuse, .63 for physical neglect, and .91 for emotional neglect (Thombs, Bernstein, Lobbestael & Arntz, 2009). A continuous variable was constructed based on the total score of each participant called “childhood adversity” (CA) leaving out the validity items.

#### 1.2.2. Parental bonding

The Parental Bonding Instrument is a self-report questionnaire of two parenting styles, Care and Overprotection, as measured by 25 items (Gordon Parker, Tupling & Brown, 1979). It is designed for mother and father separately. All items are measured on a 4-point Likert scale, ranging from “very unlikely” to “very likely.” Positive items are scored as 0 = very unlikely and 3 = very likely, while negative items are scored in reverse fashion. The “Care” subscale aims to measure facets of coldness and neglect versus affection and emotional warmth, while the “Overprotection” subscale focuses on facets of independence versus control and intrusion. Optimal parenting is resembled by high care and low overprotection, while neglectful parenting is resembled by low care and low overprotection (Craissati, McClurg & Browne, 2002). The instrument has demonstrated strong psychometric properties, including long-term temporal stability and high internal consistency (Cronbach’s α = .74–.95) (Parker, 1989; Wilhelm, Niven, Parker & Hadzi-Pavlovic, 2005). To construct a continuous measure for our analysis, we used a compound sum-score of ascending care and the inverse of ascending control, i.e., a higher sum-score means higher care and lower control, consistent with previous work (Ambruster & Witherington, 2016).

#### 1.2.3. Bullying

The Retrospective Bullying Questionnaire (RBQ) was used to assess previous experiences of bullying (Schäfer, 2004). The RBQ consists of 44 multiple choice items and short answer questions. Past experiences with victimization (physical, verbal, and indirect) are assessed, both in primary school as well as in secondary school. Single items consisting of five-point rating scales were used to measure frequency, intensity, and duration of each of the types of incidents. High test–retest reliability has been documented for the RBQ using Spearman correlation coefficients of primary school (*r* = .88) and secondary school (*r* = .87) (Schäfer, 2004), and a Cronbach’s alpha of .912 (Lund & Ross, 2021). For this analysis, in line with the previous research (Schäfer, 2004), we used a dichotomized variable for both primary and secondary school representing whether any victimization on any subscale (physical, verbal, or indirect) occurred.

### 1.3. Outcome: Neuroticism

The Dutch translation of the 12-item neuroticism scale of the Eysenck Personality Questionnaire (EPQ) was used to measure neuroticism (Sanderman, Eysenck & Arrindell, 1991). The EPQ comprises 12 questions representing nervousness, emotional lability, feelings of guilt, and low self-esteem, in a no (scored as 0) or yes (scored as 1) format. A sum score (range: 0–12) was constructed following a Dutch manual (Sanderman, Arrindell, Ranchor, Eysenck & Eysenck, 2012).

### 1.4. Genotyping

As reported previously (Pries, Klingenberg et al., 2020), genotypes of the twins and their siblings were generated on two platforms: the Infinium CoreExome-24 and Infinium PsychArray-24 kits. Quality control (QC) procedures were performed using PLINK v1.9 (Purcell et al., 2007) in both datasets separately (see Supplementary File for details).

### 1.5. PRS calculation and selection

Twelve PRS_*N*_ were calculated based on the GWAS meta-analysis result for the harmonized Neuroticism scores harmonized by item response theory (IRT) from the Genetics of Personality Consortium (GPC) (de Moor et al., 2015; van den Berg et al., 2014), as well as UK Biobank (UKB) cohort (Fry et al., 2017) (see Supplementary File for details).

The PRS_*N*_ threshold to be used in the analyses was selected after comparing candidate models with different PRS_*N*_ thresholds based on their *R* ^2^ values. As *R* ^2^ values, we used the marginal *R* ^2^ that summarizes the goodness-of-fit of a model as the proportion of explained variance by its fixed-effects terms to the total variance in the data (Nakagawa & Schielzeth, 2013; Snijders & Bosker, 2012; Xu, 2003), using the sjstats package in R (Lüdecke, 2020). For the analysis, we used PRS_*N*_ with the highest *R* ^2^ value which also was significantly associated with the outcome (*p* < .05).

### 1.6. Statistical analysis

We analyzed the association of the GPC and UKB trained PRS_*N*_, CA (Childhood Trauma Questionnaire sum score), parental bonding (separate for both parents; i.e. maternal and paternal bonding), and bullying (separately for primary and secondary school) with neuroticism (EPQ sum score) using interaction models. For the purpose of this analysis, parents and siblings were excluded. These data have a hierarchical structure due to the twin sample design, and multilevel mixed effect modeling was applied to take into account relatedness (Guo & Wang, 2002; Hunter, 2021). In keeping with an earlier publication, the hierarchical structure consisted of subjects (level 1), who were part of twin pairs (level 2) (Pries, Klingenberg et al., 2020). These multilevel mixed-effects models accounted for variability associated with each level of nesting (Carlin, Gurrin, Sterne, Morley & Dwyer, 2005; Simons et al., 2009; Snijders & Bosker, 1999). Positive skewness was observed in neuroticism. Therefore, all neuroticism scores were inverse rank normalized in accordance with previous research (Beasley, Erickson & Allison, 2009). In consideration of all these factors, we used a multilevel mixed effects model with an unstructured covariance matrix using Stata (version 15.0.49). “Mixed” or “xtmelogit” commands were used, depending whether the dependent variable was continuous or dichotomous, respectively. The independent variables (PRS, CTQ, parental bonding score, and age) were standardized (mean = 0, SD = 1).

All models were controlled for known covariates (age and sex), including adjustment for ancestry using the first two genomic principal components (PCs), in keeping with previous research (Pries, van Os et al., 2020). Interaction models included these covariates not only as additive effects but also as covariate × environment and covariate × PRS interaction terms in order to adequately control for confounding (Keller, 2014). We report results after correction; full results are shown in tables. Additionally, we tested where there was any gene–environment correlation (rGE) present in our sample for the relevant variables.

## 2. Results

### 2.1. Sample characteristics

The total sample consisted of 778 twins of which 638 provided genetic data. This final sample consisted of 202 monozygotic twin individuals and 436 dizygotic twin individuals (319 twin pairs). Sixty percentage of the sample was female, with a mean age of 17 years (range 14–34). Table 1 reports detailed socio-demographic and sample characteristics.

**Table 1.** Characteristics of samples with complete GWAS results

N (Total sample)	778
N (Completed GWAS)	638
Monozygotic	202
Dizygotic	436
Twin pairs	319

	N	Mean	SD	range
age	638	17.34	3.60	14–34
Female (%)	385 (60.34)			
Neuroticism	623	4.90	3.02	0–12
Childhood adversity	618	34.24	8.61	25–114
Maternal bonding	610	52.53	11.17	12–75
Paternal bonding	614	55.99	10.50	18–75
Bullying – primary school (% bullied)	638	17.24		
Bullying – secondary school (% bullied)	638	15.05		

### 2.2. Neuroticism’s variance explained by common SNPs

Twelve different PRS_N_ thresholds (.5 – 5 × 10^−8^) were analyzed to determine the threshold with the highest variance explained. For the GPC PRS_*N*_, the threshold of .005 explained 4.8% of the variance in neuroticism with a *p*-value of .009. The UKB PRS_*N*_ (threshold .2) explained 5.5% of the variance in neuroticism with a *p*-value of .015. There was not much heterogeneity in variance explained by the different thresholds across both sets (range 3.8% – 5.5%, as shown in Table 2). In order to explain the maximal variance, further analyses were conducted using the PRS_*N*_ at *p*-threshold .005 (GPC) and .2 (UKB).

**Table 2.** Variance in neuroticism explained by PRS_N_ in relation to phenotypical neuroticism at different PRS P-value thresholds.

	GPC		UKB	
PRS_N_	*R* ^2^	*P*-value	*R* ^2^	*P*-value
5 × 10^–8^	0.040	0.247	0.045	0.549
5 × 10^–7^	0.040	0.247	0.045	0.580
5 × 10^–6^	0.038	0.867	0.046	0.402
5 × 10^–5^	0.039	0.466	0.049	0.123
5 × 10^–4^	0.038	0.971	0.050	0.100
5 × 10^–3^ *	0.048	0.009	0.049	0.129
0.05	0.045	0.023	0.054	0.021
0.1	0.042	0.086	0.053	0.026
0.2^**†**^	0.042	0.105	0.055	0.015
0.3	0.043	0.056	0.055	0.017
0.4	0.044	0.044	0.055	0.017
0.5	0.043	0.052	0.054	0.023

*Note:* *PRS_N_ with highest R^2^ including p < 0.05 in GPC cohort. ^**†**^PRS_N_ with highest R^2^ including p < 0.05 in UKB cohort. Polygenic risk score for neuroticism (PRS_N_), genetics of personality consortium (GPC), United Kingdom Biobank (UKB).

### 2.3. Effects of PRS_*N*_, environmental factors, and interactions

Both PRS_*N*_ and environmental factors (bullying, childhood trauma, and parental bonding) were associated with neuroticism (see Table 3). The results remained significant after correction for a priori covariates (age and sex). The directions of effect for the predictors were in the hypothesized direction. Results from the analyses using both PRS_*N*_ indicated that higher neuroticism scores were associated with higher PRS_*N*_ (GPC PRS_*N*_ at .005: B .10, *p* < .01, 95% CI .03–.18; UKB PRS_*N*_ at .2: B .09, *p* = .02, 95% CI .02–.17), higher childhood trauma (B .22, *p* < .01, 95% CI .15–.28), presence of bullying (primary school: B .39, *p* < .01, 95% CI .20–.57; Secondary school: B .43, *p* < .01, 95% CI .23–.63), and lower maternal and paternal bonding scores (maternal bonding: B −.21, *p* < .01, 95% CI −.28 – −.14; paternal bonding: B −.18, *p* < .01, 95% CI −.25 – −.12).

**Table 3.** Multilevel mixed-effects model with unstructured covariance matrix of shown variables and phenotypical neuroticism as measured by EPQ.

Main effect model	Observations	*B*	95% CI	*P*-value
PRS_N_ 0.005 (GPC)	623	0.09	0.01–0.17	0.02
PRS_N_ 0.005* (GPC)	623	0.10	0.03–0.18	<0.01
PRS_N_ 0.2 (UKB)	616	0.09	0.02–0.17	0.02
PRS_N_ 0.2* (UKB)	616	0.09	0.02–0.17	0.02
Childhood adversity	752	0.20	0.13–0.27	<0.01
Childhood adversity*	752	0.22	0.15–0.28	<0.01
Maternal bonding	736	–0.19	–0.27 to –0.12	<0.01
Maternal bonding*	736	–0.21	–0.28 to –0.14	<0.01
Paternal bonding	745	–0.15	–0.22, –0.08	<0.01
Paternal bonding*	745	–0.18	–0.25 to –0.12	<0.01
Bullying – primary school	758	0.41	0.22–0.60	<0.01
Bullying – primary school*	758	0.39	0.20 to 0.57	<0.01
Bullying – secondary school	758	0.46	0.26 to 0.67	<0.01
Bullying – secondary school*	758	0.43	0.23–0.63	<0.01

*Note:* *After adjustment for age and sex. Confidence interval (CI), polygenic risk score for neuroticism (PRS_N_), genetics of personality consortium (GPC), United Kingdom Biobank (UKB), childhood adversity as measured by CTQ, maternal and paternal bonding as measured by PBI, bullying in primary and secondary school as measured by RBQ.

With respect to the interaction models, we did not find statistical interactions between PRS_*N*_ and environmental factors: childhood trauma, paternal and maternal bonding, and bullying (primary and secondary school), except solely for the interaction term of the GPC PRS_*N*_ with maternal bonding (B − .08, *p* = .04, 95% CI − .15 – −.00, see Table 4). This result did not replicate using the UKB PRS_*N*_.

**Table 4.** Multilevel mixed-effects model with unstructured covariance matrix of interaction between shown variables, PRS_N_ (GPC), and phenotypical neuroticism as measured by EPQ.

Interaction model	Interaction			
GPC	Obs.	*B*	95% CI	*P*-value
Childhood adversity	617	0.04	–0.04 to 0.13	0.29
Childhood adversity*	617	0.04	–0.04 to 0.12	0.30
Maternal bonding	609	–0.06	–0.14 to 0.01	0.09
Maternal bonding*	609	–0.08	–0.15 to –0.00	0.04†
Paternal bonding	613	–0.03	–0.10 to 0.05	0.45
Paternal bonding*	613	–0.03	–0.11 to 0.04	0.36
Bullying – primary school	623	0.08	–0.14 to 0.31	0.46
Bullying – primary school*	623	0.06	–0.16 to 0.28	0.62
Bullying – secondary school	623	–0.12	–0.35 to 0.11	0.30
Bullying – secondary school*	623	–0.17	–0.40 to 0.05	0.14
UKB				
Childhood adversity	610	–0.08	–0.17 to 0.01	0.08
Childhood adversity*	610	–0.06	–0.15 to 0.03	0.17
Maternal bonding	602	–0.05	–0.12 to 0.03	0.22
Maternal bonding*	602	–0.06	–0.14 to 0.01	0.10
Paternal bonding	606	0.01	–0.07 to 0.09	0.80
Paternal bonding*	606	0.00	–0.08 to 0.08	0.97
Bullying – primary school	616	0.05	–0.15 to 0.24	0.64
Bullying – primary school*	616	0.03	–0.17 to 0.22	0.77
Bullying – secondary school	616	0.06	–0.16 to 0.29	0.59
Bullying – secondary school*	616	0.01	–0.21 to 0.24	0.92

*Note:* *After adjustment for age and sex. ^†^Significant result with p <0.05. Confidence interval (CI), polygenic risk score for neuroticism (PRS_N_), genetics of personality consortium (GPC), United Kingdom Biobank (UKB), childhood adversity as measured by CTQ, maternal and paternal bonding as measured by PBI, bullying in primary and secondary school as measured by RBQ.

When testing for rGE, we did not find any correlation between PRS_*N*_ and environmental factors (Table 5).

**Table 5.** rGE: PRS_N_ (GPC and UKB) on environmental factors

GPC	Obs.	*B*	95% CI	*P*-value
Childhood adversity	618	0.02	–0.06 to 0.102	0.62
Maternal bonding	610	–0.02	–0.11 to 0.06	0.58
Paternal bonding	614	–0.04	–0.13 to 0.04	0.32
Bullying – primary school	638	0.01	–0.29 to 0.31	0.93
Bullying – secondary school	638	0.16	–0.18 to 0.51	0.35
UKB				
Childhood adversity	611	0.06	–0.03 to 0.14	0.19
Maternal bonding	603	–0.07	–0.16 to 0.01	0.09
Paternal bonding	607	–0.07	–0.15 to 0.01	0.10
Bullying – primary school	631	0.17	–0.12 to 0.47	0.25
Bullying – secondary school	631	0.01	–0.32 to 0.35	0.93

*Note:* Confidence interval (CI), polygenic risk score for neuroticism (PRS_N_), genetics of personality consortium (GPC), and United Kingdom Biobank (UKB). ADDIN

## 3. Discussion

To the best of our knowledge, there has been sparse research investigating gene–environment interactions underlying the phenotype of neuroticism with the use of polygenic risk scores (Lehto et al., 2019; Werme et al., 2021). In a unique adolescent and young adult twin sample, we showed that the PRS_*N*_, as well as parental bonding, exposure to bullying, and childhood trauma, were associated with phenotypic expression of neuroticism. However, there was no evidence for interaction between PRS_*N*_ and any of the environmental factors.

Both PRS_*N*_ were associated with neuroticism and explained 4.8% (GPC, .005 threshold) and 5.5% (UKB, 0.2 threshold) of the variance in this population, which is within the range of other publications (de Moor et al., 2015; Nagel et al., 2018; Okbay et al., 2016; Werme et al., 2021). The estimated variances explained in these publications ranges from .011% up to 15%. PRS_*N*_ explained up to 4.2% of variance in phenotypical neuroticism in the UK Biobank cohort (UKB) (Nagel et al., 2018). In the GPC cohort, this was 15% in the two target datasets (de Moor et al., 2015). Although Okbay et al. used four cohorts (including UKB and GPC cohorts), they only found around .7% variance explained (Okbay et al., 2016). These findings suggest that neuroticism has a highly complex genetic background including both common and rare variants.

When considering early and later life stressful environmental factors, we found an association between neuroticism and parenting style. Our findings suggest that affectionate and warm parenting styles without controlling or intrusive behavior by the parents are associated with a decline in neuroticism, which can be explored further as a potential net benefit in future studies. On the other hand, having been bullied, or having experienced any form of childhood abuse or neglect was associated with an increase in neuroticism.These findings are largely consistent with previous research (Huppert, Abbott, Ploubidis, Richards & Kuh, 2010; Jeronimus, Ormel, Aleman, Penninx & Riese, 2013; Ono et al., 2017; Seki et al., 2020; Takahashi et al., 2017). By investigating positive and negative life events with the 20-item List of Threatening Experiences, a study found that distant (occurring on average a year before follow-up) negative life events were associated with increased neuroticism, while distant positive life events were associated with decreased neuroticism (Jeronimus et al., 2013). The study also showed a moderating effect of childhood adversity. Specifically, those who experienced childhood adversity had higher baseline neuroticism and less increase in neuroticism after distant negative life events but more decrease in neuroticism after distant positive life events. Our results confirm that childhood adversity is associated with an increased expression of neuroticism, whereas an optimal parenting style (e.g., high care and low overprotection) is associated with decreased neuroticism. Several studies found that optimal parenting had a decreasing effect on neuroticism (Ono et al., 2017; Takahashi et al., 2017). Furthermore, neuroticism can be considered a mediator of the effect of the quality of parenting on depressive symptoms (Ono et al., 2017). Others showed that high perceived job stress and stress response in adult employees are still indirectly influenced by both parental overprotection and care via neuroticism, underscoring the long-term effects of suboptimal parenting in development (Seki et al., 2020). When assessing mental well-being in a more general sense, it was found that a high care and low overprotection parenting style were associated with mental well-being that was largely mediated by personality (Huppert et al., 2010). These findings emphasize the societal impact of optimal parenting and lower neuroticism on mental well-being.

Only a few studies have thus far utilized PRS_*N*_ in GxE models. In a recent study, Lehto et al. investigated the gene–environment interaction between PRS_*N*_ and early-life stress using raised together or apart as a proxy for childhood adversity (Lehto et al., 2019). They found that the PRS_*N*_ only had a significant association with the expression of depression in reared-together twins in their cohort of older individuals, whereas the results showed a trend toward statistical significance for neuroticism. Their G  × E analyses revealed considerably stronger effect of PRS_*N*_ on neuroticism in the reared-together twins, suggestive of heterogeneity in neuroticism development depending on childhood adversity. They showed an interaction between PRS_*N*_ and rearing status for depressive symptoms and a similar pattern for neuroticism and loneliness. More recently, a comprehensive genome-wide gene–environment interaction study showed that although there was some benefit to this approach, interaction effects appeared to not predict much more variance in phenotypical neuroticism beyond main effects (Werme et al., 2021). We measured childhood adversity with a retrospective questionnaire and showed a significant association with neuroticism without any interaction with the PRS_*N*_. Some of these differences may be explained due to the use of different training datasets to calculate PRS. We used the GPC as well as UKB datasets to estimate PRS_*N*_, whereas Lehto et al. used a pooled data including the UKB. It may be likely that minor variations in estimating PRS_*N*_ may result in differences in the final results. Additionally, differences in the definition of childhood adversity might explain parts of the observed difference in results.

Finally, we demonstrated that the exposure to bullying during childhood was associated with higher neuroticism scores and that this exposure did not show a statistical interaction with PRS_*N*_. In this regard, findings from the ALSPAC cohort in over 5000 participants showed no association between the risk of exposure to bullying and PRS_*N*_ (Schoeler et al., 2019). Previous reports showed an association between bullying and neuroticism (Rosta & Aasland, 2018), as well as an association between neuroticism and being an aggressor (Pabon-Carrasco et al., 2019). Furthermore, there has been plenty of research on the link between neuroticism and depression (Christensen & Kessing, 2006), but research on the interaction between neuroticism and exposure to bullying yields some contradictory results. A longitudinal study showed that although neuroticism predicted both depression and social anxiety, no significant interactions were evident between neuroticism and bullying victimization (Calvete, Orue & Gamez-Guadix, 2016).

Some strengths and limitations of our study need to be mentioned. A major strength of our research was the sample that consisted of a mixed-age group ranging from 14 to 34 years of age. In personality research, it is essential to capture early arising gender typical manifestations of neuroticism, which usually manifests earlier in women than in men (as early as 12–14 years of age) (De Bolle et al., 2015). Some differences and contrasts have even been reported earlier (Borghuis et al., 2017; McCrae, Costa & Martin, 2005; McCrae et al., 2002). However, early variation is often overlooked in predominantly older cohorts (Lehto et al., 2019). Our young sample is suited to capture this early variation but may be underpowered to detect gene–environment interaction. The marginally significant finding for the interaction between PRS_*N*_ (GPC) and maternal bonding, which did not replicate with PRS_*N*_ (UKB), possibly indicates that some interactions might have been detected if our sample had been larger. Therefore, further research using larger cohorts is warranted. One could also note that a twin population may not be considered a complete representation of the general population, thereby making generalizations toward other populations difficult. In GWAS, the population structure has an impact on polymorphism distributions varying in regard to neuroticism as well. For instance, a study in older adults with African and White European ancestries posited that existing PRS were derived from mostly or exclusively White European samples and this limits their applicability in other ancestries (Assari et al., 2020). Other research showed that it might be possible to predict across ancestries in regards to neuroticism (Docherty et al., 2016). Our sample was a very homogenous cohort consisting of predominantly individuals of European ancestry. Therefore, we were able to apply PRS_*N*_ conveniently. However, it must be noted our findings may not be generalizable to other ethnic ancestries. Another limitation is the general caveat of self-reporting bias including social desirability, especially relevant when assessing childhood adversity, and recall bias. Given our interest in neuroticism, a phenotype measuring a propensity for negative emotion, it might be possible that people with high neuroticism might have a heightened sense of the negative in their life and tend to report subjectively intensified childhood adversities in retrospect when responding to retrospective measures. Although we cannot preclude the influence of subjective experience of adversity that might be due to neuroticism as a whole phenotype, our assessment of gene–environment correlation (rGE) shows that at least the genomic portion of neuroticism does not seem to be correlated with childhood adversity. Further research should include the use of independent raters, objective indicators, and prospective designs to generate additional robust evidence. An additional direction to take would be broadening the assessed environmental factors, ideally aiming to assess the entire exposome using an agnostic approach (Lin et al., 2022).

In conclusion, we showed that PRS_*N*_, as well as parental bonding, bullying, and childhood trauma, were independently associated with the phenotypic expression of neuroticism, but there was no evidence for gene–environment interaction. Although the etiology of neuroticism including highly polygenic background and various early environmental insults is slowly disentangled, there is still no clear plausible mechanistic pathway from genetic and environmental variation via intermediate phenotypes toward clinical expressions of psychopathology.

## Data Availability

The data that support the findings of this study are available from the corresponding author upon reasonable request under the condition of the approval of the TwinssCan steering committee.

## Supplementary material

For supplementary material accompanying this paper visit https://doi.org/10.1017/pen.2023.2. click here to view supplementary material
