# Supplementary material for: Gene–environment interaction study on the polygenic risk score for neuroticism, childhood adversity, and parental bonding
Source: Personal Neurosci. 2023 Aug 4;6:e5. doi: 10.1017/pen.2023.2 (PMC10725776; doi:10.1017/pen.2023.2)
Supplement: Supplementary file 1 [file S2513988623000020sup001.docx]

**Supplementary Methods**

*2.4. Genotyping*

Single nucleotide polymorphisms (SNPs) and participants with call rates below 95% and 98%, respectively, were removed. A strict SNP QC only for subsequent sample QC steps was then conducted. This involved a minor allele frequency (MAF) threshold >10% and a Hardy–Weinberg equilibrium (HWE) *P*‐value >10^−5^, followed by linkage disequilibrium (LD)‐based SNP pruning (*R*^2^ < 0.5). This resulted in ~58K SNPs to assess sex mismatch (*n* = 8), heterozygosity [*F* < mean - 5× the standard deviation (SD), *n* = 3], homozygosity (*F* >mean + 5× SD), and relatedness by pairwise identity by descent (IBD) values (monozygotic:$\hat{p}<0.9$ , dizygotic and full siblings: : $\hat{p}>0.65$  or $\hat{p}< 0.35$, *n* = 5). The ancestry‐informed principal component analyses (PCA) were conducted by EIGENSTRAT (Price et al., 2006). The ethnic outliers of which the first 4 PCs diverged >10× SD from Utah residents with Northern and Western European ancestry from the CEPH collection (CEU) and Toscani in Italia (TSI) samples (*n* = 5), and >3× SD of the TwinssCan samples (*n* = 7) were excluded ( see also supplementary figure 1 and supplementary table 1). After removing these subjects, a regular SNP QC was performed (SNP call rate >98%, HWE *P* > 1e‐06, MAF > 1%, and strand ambiguous SNPs and duplicate SNPs were removed).

The two QCed datasets were imputed on the Michigan server (Das et al., 2016) using the HRC r1.1 2016 reference panel with European samples after phasing with Eagle v2.3. Postimputation QC involved removing SNPs with imputation quality (*R*^2^) < 0.8, with a MAF < 0.01, SNPs that had a discordant MAF compared to the reference panel (MAF difference with HRC reference > 0.15), as well as strand ambiguous AT/CG SNPs and multi‐allelic SNPs. The two chips were merged, and an additional check for MAF > 0.01, HWE *P*> 1e‐06 was executed, which resulted in 3,407,392 SNPs for 688 individuals. The general imputation quality is shown in Supplementary Figure 2.

*2.5. PRS Calculation and selection*

The GPC meta-analysis results were based on 29 discovery cohorts, with 21 cohorts from Europe, 6 from the Unites States and 2 from Australia. One cohort (ALSPAC) provided subjects with a mean age as low as 13.8 years (SD=0.21). All participants were of European descent. The total GPC sample size was 63,661 for the GWA meta-analysis. The UKB is a population-based cohort of over 500,000 participants recruited in the United Kingdom (UK) between 2006 and 2010. Individuals were aged 40–69 years (mean age 56.52) and lived near 22 assessment centers in England, Wales, and Scotland (Fry et al., 2017). Due to the differences in cohort size the PRS_N_ trained on the UKB was expected to outperform the PRS_N_ trained on the GPC when looking at the total number of variants passing the threshold of genomewide significance. The caveat is that the age distribution in the UKB cohort is relatively old (mean age 56.52) thus not representing our relatively young sample (mean age 17.34). All though neuroticism is relatively stable across time, there are age effects. Thus we report both results. To calculate all PRS_N_, the beta-values, effective allele, and P-values were extracted from all summary statistics. Insertions and deletions, ambiguous SNPs, SNPs with a MAF < 0.01 and/or imputation quality R2 < 0.9, as well as SNPs located in complex-LD regions and long-range LD regions (Price et al., 2008) (see Supplementary table 2) were excluded. Overlapping SNPs between each neuroticism GWAS (training dataset), 1000 genomes (reference), and our TwinssCan dataset (target) were selected. These SNPs were clumped in two rounds using PLINK’s clump function (round 1: --clump-kb 250 --clump-r2 0.5; round 2: --clump-kb 5000 --clump-r2 0.2). The numbers of alleles for PRS_N_ calculation are listed in Supplementary Table 3. Odds ratios for autosomal SNPs reported in the neuroticism summary statistics were log-converted into beta values. PRS_N_ were calculated using PLINK’s score function (Purcell et al., 2007) at the following P-value thresholds: 5 x 10^-8^, 5 x 10^-7^, 5 x 10^-6^, 5 x 10^-5^, 5 x 10^-4^, 5 x 10^-3^, 0.05, 0.1, 0.2, 0.3, 0.4 and 0.5.

**References**

Das, S., Forer, L., Schonherr, S., Sidore, C., Locke, A. E., Kwong, A., . . . Fuchsberger, C. (2016). Next-generation genotype imputation service and methods. *Nat Genet, 48*, 1284-1287. doi:10.1038/ng.3656

Fry, A., Littlejohns, T. J., Sudlow, C., Doherty, N., Adamska, L., Sprosen, T., . . . Allen, N. E. (2017). Comparison of Sociodemographic and Health-Related Characteristics of UK Biobank Participants With Those of the General Population. *Am J Epidemiol, 186*, 1026-1034. doi:10.1093/aje/kwx246

Price, A. L., Patterson, N. J., Plenge, R. M., Weinblatt, M. E., Shadick, N. A., & Reich, D. (2006). Principal components analysis corrects for stratification in genome-wide association studies. *Nat Genet, 38*, 904-909. doi:10.1038/ng1847

Price, A. L., Weale, M. E., Patterson, N., Myers, S. R., Need, A. C., Shianna, K. V., . . . Reich, D. (2008). Long-range LD can confound genome scans in admixed populations. *Am J Hum Genet, 83*, 132-135; author reply 135-139. doi:10.1016/j.ajhg.2008.06.005

Purcell, S., Neale, B., Todd-Brown, K., Thomas, L., Ferreira, M. A., Bender, D., . . . Sham, P. C. (2007). PLINK: a tool set for whole-genome association and population-based linkage analyses. *Am J Hum Genet, 81*, 559-575. doi:10.1086/519795

# Supplementary Figure 1

# *The first and second principal component of TwinssCan data (with identified ethnic outliers) along with hapmap3 populations.*

# Supplementary Table 1

# *Eigenvalues and proportion variance explained for the first 20 PCs from PCA analyses.*

| **PCs** | **model 1: PCA with TwinssCan and hapmap3 population** | | | **model 2: PCA with TwinssCan cohort** | | |
| --- | --- | --- | --- | --- | --- | --- |
|  | **Eigenvalues** | **proportion variance** | **accumulated variance** | **Eigenvalues** | **proportion variance** | **accumulated variance** |
| PC1 | 164.21 | 0.50 | 0.51 | 3.04 | 0.07 | 0.07 |
| PC2 | 101.01 | 0.31 | 0.82 | 2.75 | 0.06 | 0.12 |
| PC3 | 8.52 | 0.03 | 0.84 | 2.55 | 0.05 | 0.18 |
| PC4 | 7.80 | 0.02 | 0.87 | 2.48 | 0.05 | 0.23 |
| PC5 | 7.15 | 0.02 | 0.89 | 2.45 | 0.05 | 0.28 |
| PC6 | 2.77 | 0.01 | 0.90 | 2.43 | 0.05 | 0.34 |
| PC7 | 2.74 | 0.01 | 0.90 | 2.42 | 0.05 | 0.39 |
| PC8 | 2.69 | 0.01 | 0.91 | 2.36 | 0.05 | 0.44 |
| PC9 | 2.59 | 0.01 | 0.92 | 2.33 | 0.05 | 0.49 |
| PC10 | 2.49 | 0.01 | 0.93 | 2.21 | 0.05 | 0.54 |
| PC11 | 2.47 | 0.01 | 0.94 | 2.20 | 0.05 | 0.58 |
| PC12 | 2.43 | 0.01 | 0.94 | 2.19 | 0.05 | 0.63 |
| PC13 | 2.39 | 0.01 | 0.95 | 2.18 | 0.05 | 0.68 |
| PC14 | 2.35 | 0.01 | 0.96 | 2.17 | 0.05 | 0.72 |
| PC15 | 2.35 | 0.01 | 0.97 | 2.17 | 0.05 | 0.77 |
| PC16 | 2.31 | 0.01 | 0.97 | 2.16 | 0.05 | 0.82 |
| PC17 | 2.26 | 0.01 | 0.98 | 2.15 | 0.05 | 0.86 |
| PC18 | 2.26 | 0.01 | 0.99 | 2.15 | 0.05 | 0.91 |
| PC19 | 2.24 | 0.01 | 0.99 | 2.15 | 0.05 | 0.95 |
| PC20 | 2.22 | 0.01 | 1.00 | 2.15 | 0.05 | 1.00 |

# Supplementary Figure 2

# *1a. Correlation of SNPs MAF from chip1 dataset with the reference MAF.*


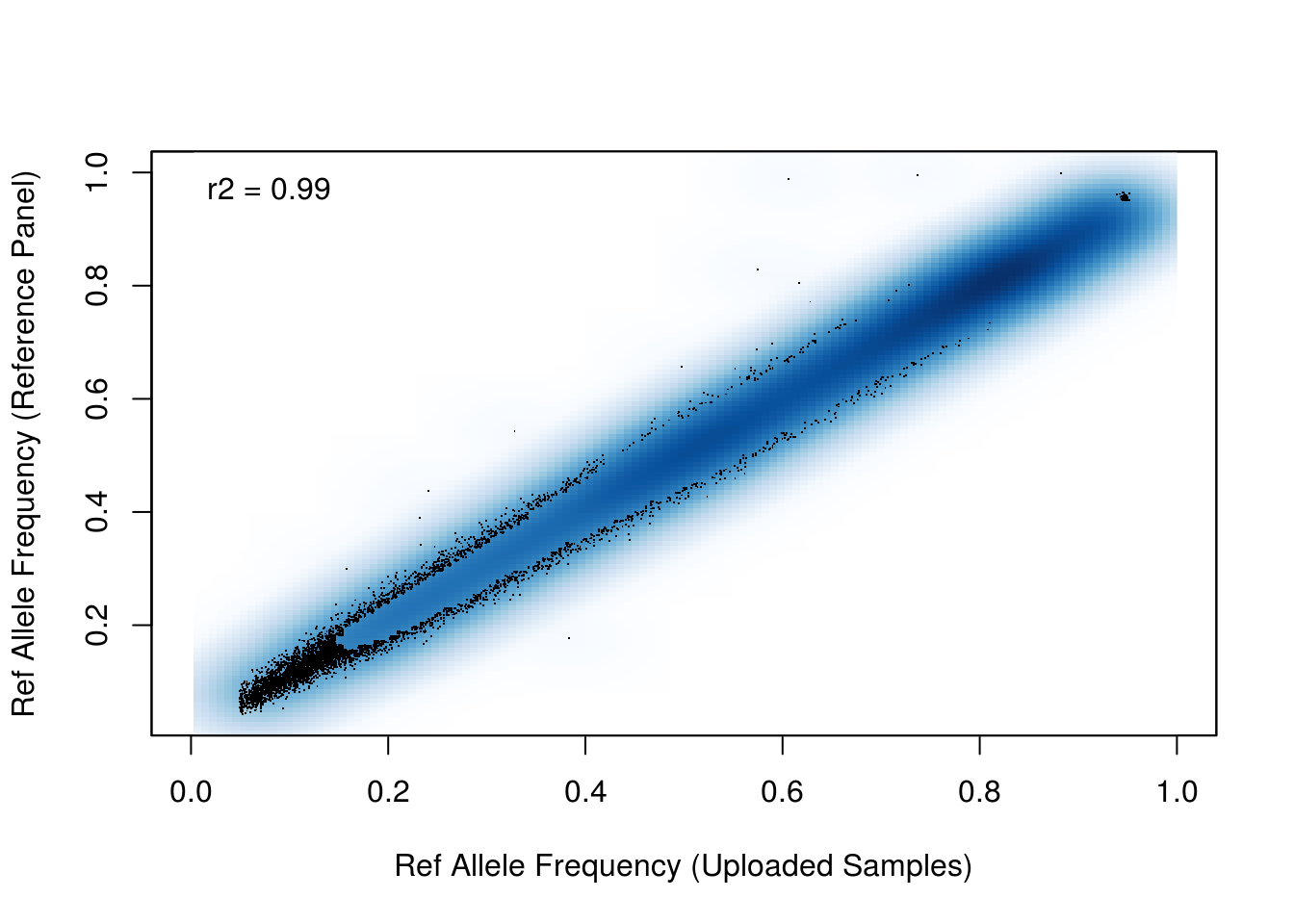


*1b. Correlation of SNPs MAF from chip 2 dataset with the reference MAF.*
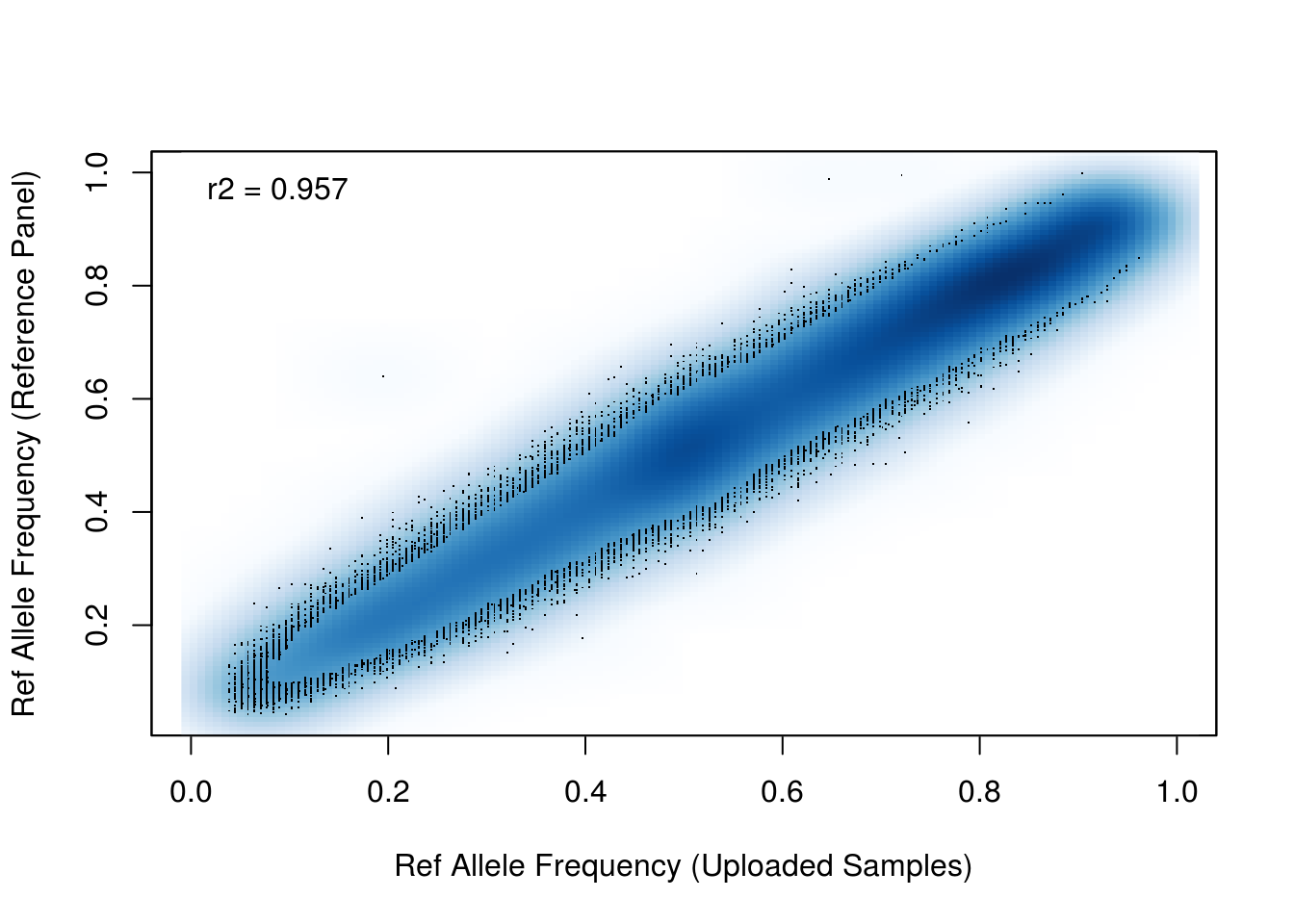


**Supplementary Table 2**

*20 Complex-LD regions and long-range LD regions which were excluded from PRS analysis.*

| **Chromosome** | **Base pair position**  **(start point to end point)** |
| --- | --- |
| 1 | 48000000-52000000 |
| 2 | 86000000-100500000 |
| 2 | 183000000-190000000 |
| 3 | 47500000-50000000 |
| 3 | 83500000-87000000 |
| 5 | 44500000-50500000 |
| 5 | 129000000-132000000 |
| 6 | 25500000-33500000 |
| 6 | 57000000-64000000 |
| 6 | 140000000-142500000 |
| 7 | 55000000-66000000 |
| 8 | 8000000-12000000 |
| 8 | 43000000-50000000 |
| 8 | 112000000-115000000 |
| 10 | 37000000-43000000 |
| 11 | 87500000-90500000 |
| 12 | 33000000-40000000 |
| 20 | 32000000-34500000 |
| 8 | 8135000-12000000 |
| 17 | 40900000-45000000 |

*Note:* Polygenic risk score (PRS).

#

**Supplementary Table 3**

*The number of alleles used for PRS calculation for the TwinssCan data at different P-value thresholds using the different cohorts.*

| Level | *P*-value threshold | Number of alleles | |
| --- | --- | --- | --- |
|  |  | GPC | UKB |
| 1 | 5X10-8 | 2 | 136 |
| 2 | 5X10-7 | 2 | 226 |
| 3 | 5X10-6 | 8 | 510 |
| 4 | 5X10-5 | 102 | 1194 |
| 5 | 5X10-4 | 674 | 3480 |
| 6 | 5X10-3 | 4998 | 11764 |
| 7 | 0.05 | 8790 | 45992 |
| 8 | 0.1 | 29736 | 70322 |
| 9 | 0.2 | 43501 | 106716 |
| 10 | 0.3 | 76324 | 135028 |
| 11 | 0.4 | 96144 | 158396 |
| 12 | 0.5 | 111594 | 177738 |

*Note: Polygenic risk score (PRS ).*
